# Exploring the causal relationship between vitiligo and psoriasis: a bidirectional Mendelian randomization analysis

**DOI:** 10.1007/s00403-025-04102-4

**Published:** 2025-03-29

**Authors:** Zhengxing Xu, Chao Yang, Xuehui Gan, Peijing Yan, Changfeng Xiao, Yunli Ye, Xia Jiang

**Affiliations:** 1 School of Public Health, Southwest Medical University, No. 1 Section 1, Xianglin Road, Longmatan District, Luzhou, 646000 Sichuan China; 2 Clinical Research Center, Sichuan Provincial People’s Hospital, University of Electronic Science and Technology of China, Chengdu, China; 3 Department of Epidemiology and Biostatistics, West China School of Public Health and West China Fourth Hospital, Sichuan University, No.16, Section 3, South Renmin Road, Wuhou District, Chengdu, 610041 China; 4 Department of Nutrition and Food Hygiene, West China School of Public Health and West China Fourth Hospital, Sichuan University, Chengdu, China; 5 Department of Clinical Neuroscience, Center for Molecular Medicine, Karolinska Institutet, Solna, Stockholm, Sweden

**Keywords:** Vitiligo, Psoriasis, Causal relationship, Mendelian randomization

## Abstract

Observational studies have demonstrated an association between vitiligo and psoriasis. However, to date, the causal nature of this association remains uncertain. The objective of this study was to investigate the potential bidirectional causal relationship between vitiligo and psoriasis by employing a bidirectional two-sample Mendelian randomization (MR) approach. We utilized summary statistics obtained from the genome-wide association study (GWAS) conducted in European ancestry for vitiligo (*N* = 44,266) and psoriasis (*N* = 373,338). We first performed univariate MR analysis to detect potential bidirectional causality between vitiligo and psoriasis. Then, for directions in which univariate MR confirmed a causal relationship, we further conducted multivariate MR analysis to investigate independent causal effects on the outcome considering exposure to confounders. The bidirectional two-sample MR analysis showed genetic liability to vitiligo was significantly associated with an increased risk of psoriasis (*OR* = 1.094, 95% *CI*: 1.052, 1.138), but there was no significant association between genetic liability to psoriasis and risk of vitiligo (*OR* = 1.176, 95% *CI*: 0.915, 1.511). For the vitiligo to psoriasis direction, multivariate MR adjusting for smoking, drinking, body mass index, and rheumatoid arthritis showed the presumed causality was despite attenuated (*OR* = 1.060, 95% *CI*:1.035, 1.085), and remained statistically significant. Our study suggests that vitiligo is a causal risk factor for psoriasis, but the reverse may not be true. It is emphasized by the evidence from this study that enhanced early screening for psoriasis among patients with vitiligo may help to reduce the incidence of psoriasis.

**Supplementary Information:**

The online version contains supplementary material available at 10.1007/s00403-025-04102-4.

## Introduction

Vitiligo and psoriasis are two refractory autoimmune skin diseases commonly characterized by dysregulation of immune function and the infiltration of inflammatory cells in skin lesions [1, 2]. It is estimated that about 0.5-2% of the general population worldwide has vitiligo, and 0.51–11.43% has psoriasis [3, 4]. In clinical practice, vitiligo and psoriasis share similar pathological features (e.g., skin plaques, neuropeptide involvement, lack of specific autoantibodies, and Koebner’s phenomenon) [5] and are frequently reported to coexist [6], suggesting a potential link between the two disorders. Thus, elucidating the association between vitiligo and psoriasis may hold significant public health implications for the prevention and management of both conditions and their complications.

The relationship between vitiligo and psoriasis has been partially investigated in several previous observational epidemiologic studies, however the reported results are not entirely consistent. Some studies have shown a positive correlation [7–9], while others have shown no effect [9, 10]. In addition, several studies also explored a possible bidirectional association between vitiligo and psoriasis [11, 12]. However, these prior observational studies are limited in their ability to infer causality due to the potential for bias from unmeasured or unknown confounders and reverse causality [13]. It is unclear whether there is a causal association between vitiligo and psoriasis and the direction of the causal association.

In recent years, Mendelian randomization (MR), a novel statistical approach using genetic variants [single nucleotide polymorphisms (SNPs)] as instrumental variables (IVs), has been increasingly used to estimate causal inferences between exposures and outcomes [14]. MR is based on the random assignment of genetic variants during meiosis, thus lowering the risk of confounding or reverse causation [15]. Indeed, a recent MR study has preliminarily assessed a bidirectional causal relationship between vitiligo and psoriasis but reported non-significant bidirectional causal association [16]. However, this study (1) used a small sample size of genome-wide association studies (GWASs) summary data and included a limited number of IVs, and thus may lack sufficient statistical power to detect potential causal associations, leading to false-negative results; and (2) did not consider the influence of some important confounders (e.g., well-established risk factors for autoimmune disorders, such as smoking, drinking, etc. [17]) on the causal association between vitiligo and psoriasis, failing to accurately reveal the potential true causal effect.

Therefore, in this study, we utilized an enlarged sample size of summary statistics of vitiligo and psoriasis GWASs with an extended MR analytical strategy to investigate the potential bidirectional causality between the genetically predicted risk of vitiligo with psoriasis and the genetically predicted risk of psoriasis with vitiligo.

## Materials and methods

### Study design

**Fig. 1:**
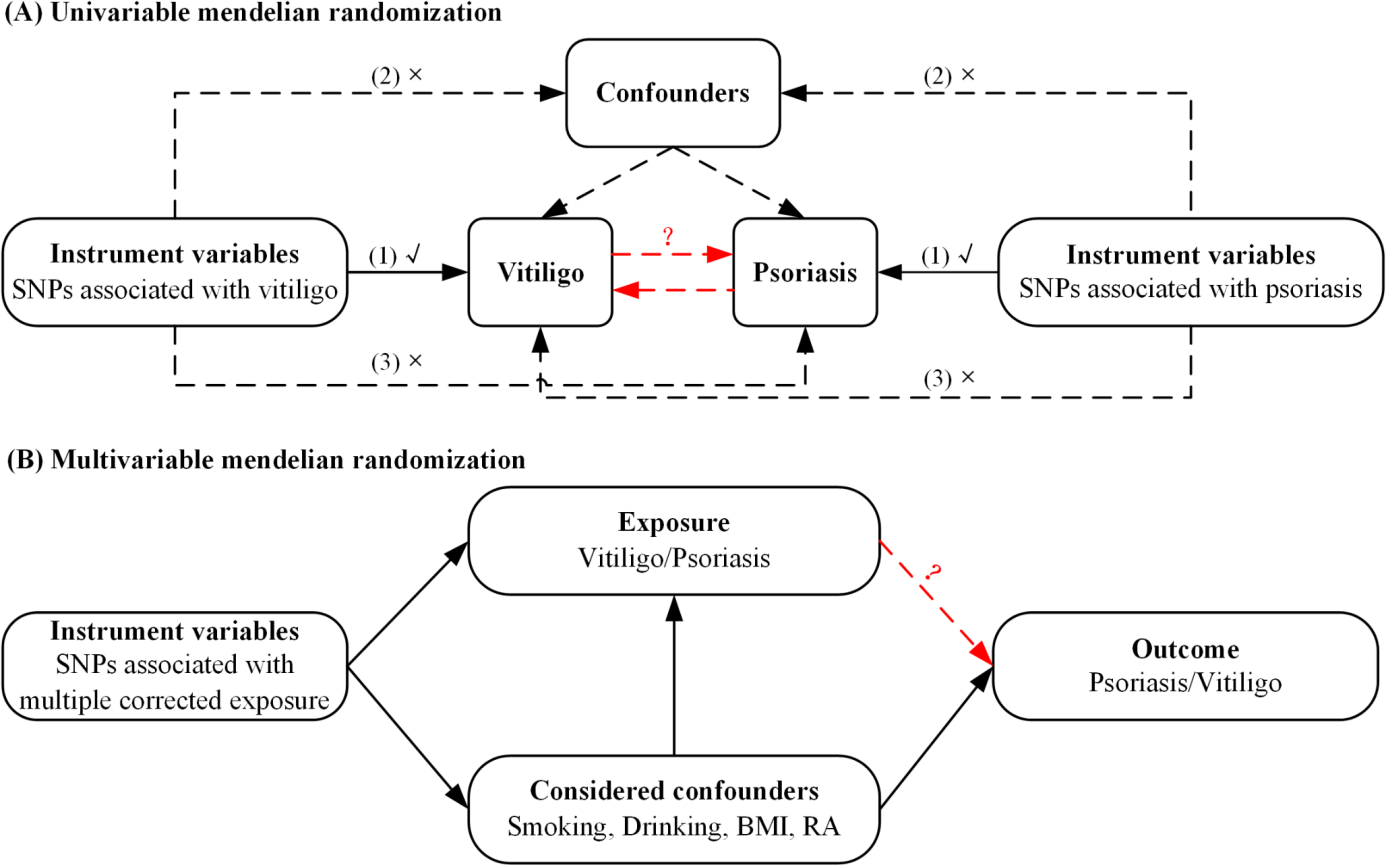
Schematic diagram of the overall design in this study. (**A**) Univariable mendelian randomization. Three core assumptions of mendelian randomization: (1) the IV is associated with the exposure of interest, (2) there is no association between IVs and confounders of the exposure–outcome relationship, and (3) the IV affects the outcome solely through its effect on the exposure. (**B**) Multivariable mendelian randomization. Abbreviations: SNP = Single-nucleotide polymorphism, BMI = Body mass index, RA = Rheumatoid arthritis

The general design of this study is shown in Fig. 1. First, we performed twice (forward and reverse) univariable MR analyses to investigate the bidirectional association between vitiligo and psoriasis. The forward MR analyses considered vitiligo as the exposure and psoriasis as the outcome to assess the causal effect of vitiligo on psoriasis, whereas reverse MR analyses of psoriasis as the exposure and vitiligo as the outcome to assess the reverse causal effect of psoriasis on vitiligo. Second, for direction(s) in which univariable MR suggested a significant causal association, we further performed multivariable MR to explore independent causal effects of exposure on outcomes by adjusting for potential horizontal pleiotropy acted through confounders [18].

### Data sources

We used data from publicly available GWASs summary data based on European ancestry. As this study was a secondary analysis of existing data, no additional ethical approval or informed consent from participants was required. Detailed information on these original GWASs data is shown in Table 1. The GWAS statistics for vitiligo were derived from the GWAS Catalog (GCST004785), comprising 44,266 individuals of European ancestry (4,680 cases and 39,586 controls) [19]. Of these, vitiligo cases were determined based on strict clinical criteria for diagnosis of generalized vitiligo. For psoriasis, the GWAS statistics were derived from data published by the FinnGen consortium (9th release), including 373,338 individuals of European ancestry (9,267 cases and 364,071 controls) [20, 21]. The definition of psoriasis cases was based on the International Classification of Diseases, Tenth Revision codes (L40-L45).

**Table 1:** Detailed information of the genome-wide association studies used in this study

Phenotypes	No. of SNPs	Sample size	Ethnicity	*R*^2^ (%) ^a^	F-statistic ^b^	PMID
**Exposure/Outcome**						
Vitiligo	48	(4,680 cases / 39,586 controls)	European	17.40	216.64	27,723,757
Psoriasis	32 ^c^	(9,267 cases / 364,071 controls)	European	0.79	92.80	36,653,562
**Confounders**						
Smoking	378	1,232,091	European	2.30	76.71	30,643,251
Drinking	99	941,280	European	0.20	19.05	30,643,251
BMI	670	806,834	European	5.60	71.38	30,239,722
RA	57	97,173	European	3.62	63.99	36,333,501

^a^*R*^*2*^ was obtained based on the original GWAS or calculated based on the following equation: *R*^*2*^ = *2 × β*^*2*^ *× MAF×(1-MAF)/(2 × β*^*2*^ *× MAF×(1-MAF) + 2se(β)*^*2*^*×N×MAF×(1-MAF)).* Of these, *β* is the effect size; *MAF* is the minor allele frequency; *se(β)* is the standard error of effect size. ^b^*F*-statistic was obtained based on the following equation: *F = R*^*2*^*(N − k−1) / k (1 − R*^*2*^*).* Of these, *R*^2^ is the proportion of variance in the phenotype explained by genetic variants; *N* is the sample size; *k* is the number of instruments. ^*c*^ SNP for psoriasis was obtained based on the “ld_clump”’ function (clump_kb = 10,000, clump_r2 = 0.001). Abbreviations: SNP = Single-nucleotide polymorphism, *R*^*2*^ = Proportion of variance in the phenotype explained by genetic variants, BMI = Body mass index, RA = Rheumatoid arthritis.

Additionally, to assess the independent causal effect of exposure on outcomes, this study also considered several potential confounders that may influence the association between vitiligo and psoriasis based on prior literature [17, 22–24], including smoking, drinking, body mass index (BMI), and rheumatoid arthritis (RA) among common autoimmune diseases. For smoking and drinking, the GWAS statistics were available from the GWAS & Sequencing Consortium of Alcohol and Nicotine Use, with 1,232,091 individuals for smoking initiation and 941,280 individuals for drinks per week [25]. For BMI, the GWAS statistics were obtained from the UK Biobank and Genetic Investigation of Anthropometric Traits consortium, totalling 806,834 individuals [26]. For RA, the GWAS statistics were derived from a meta-analysis of 25 European cohorts totaling 97,173 individuals [27].

### Selection of IVs

We extracted genome-wide significant and independent (i.e., no linkage disequilibrium) genetic variants from the original GWASs as IVs for the exposure phenotype. For vitiligo, the original GWAS identified 48 independent and genome-wide significant (*P* < 5 × 10^− 8^) genetic variants [19]. Among these 48 SNPs, 43 were found in GWAS of psoriasis and were determined as IVs for vitiligo (Supplementary Table 1). For psoriasis, since the original GWAS did not report significantly associated genetic variants, we adopted the “ld_clump” function (clump_kb = 10,000, clump_r2 = 0.001) and identified 31 independent SNPs. Among these 31 SNPs, 25 were identified in GWAS of vitiligo and were thus determined as IVs for psoriasis (Supplementary Table 2). For confounders, we also extracted independently significant SNPs as IVs, including 378 SNPs for smoking, 99 SNPs for drinking, 670 SNPs for BMI, and 57 SNPs for RA.

Due to the bias of weak instruments can lead to misestimation of causal effects. We thus assessed the strength of the IVs by calculating the *F*-statistic through the following equation: *F = R*^*2*^*(N − k−1) / k (1 − R*^*2*^) [28]. Of these, *R*^2^ is the proportion of variance in the phenotype explained by genetic variants; *N* is the sample size; *k* is the number of instruments. Typically, an *F*-statistic > 10 indicates a strong instrument and sufficient strength to ensure the validity of IV methods [28].

### Statistical analysis

#### Univariable MR analysis

A bidirectional two-sample univariable MR analysis was conducted to detect putative bidirectional causal effects of vitiligo on psoriasis and psoriasis on vitiligo. The fixed-effects or random-effects inverse-variance weighted (IVW) method was applied as the primary approach [29]. Complementary to IVW, MR-Egger regression [30] and weighted median methods [31] were adopted as alternative approaches to examine the robustness and consistency of results under relaxed model assumptions. The heterogeneity among genetic variants was assessed by employing Cochran’s Q test. Meanwhile, the presence of directional pleiotropy among these genetic variants was evaluated using MR-Egger intercepts. A causal estimate was deemed significant if it demonstrated significance (*P*-value < 0.05) in the IVW method and exhibited directional consistency in both MR-Egger regression and weighted median approach [32].

Several sensitivity analyses were conducted to validate the three core assumptions of MR: (1) the IV is associated with the exposure of interest, (2) there is no association between IVs and confounders of the exposure–outcome relationship, and (3) the IV affects the outcome solely through its effect on the exposure [33]. First, we excluded IVs with strand ambiguity that were palindromic. Second, we excluded pleiotropic IVs associated with potential confounding traits as confirmed by the GWAS Catalog. Third, we performed MR-pleiotropy residual sum and outlier (MR-PRESSO) analysis, which detects outlier IVs and re-evaluates the causal effect after removing outliers. Finally, we conducted a leave-one-out analysis, removing each IV individually, and performed IVW based on the remaining IVs to determine if a single SNP drove the causal signal. In addition, mRnd (http://cnsgenomics.com/shiny/mRnd/), an online web tool, was employed to perform statistical power calculations [34].

#### Multivariable MR analysis

For direction(s) in which univariable MR suggested a significant causal association, we further performed an IVW-based multivariable MR analysis to explore the independent causal effects of the exposure on the outcomes by single and simultaneous adjusting smoking, drinking, BMI, and RA. Considering that IVs with confounders may overlap or correlate with exposed IVs, we thus removed SNPs in linkage disequilibrium (r2 > 0.001) by applying the “ld_clump” function (clump_kb = 500, clump_r2 = 0.001) to obtain independent SNPs for analysis.

All analyses were conducted using the “TwoSampleMR”, “MR-PRESSO”, or “Mendelian randomization” package in R version 4.0.5. The effect estimates are reported as odds ratio (*OR*) and its corresponding 95% confidence intervals (*CIs*). Two-sided tests were employed for all tests, with a significance level of *P* ≤ 0.05.

## Results

### The causal effect of vitiligo on psoriasis

**Fig. 2:**
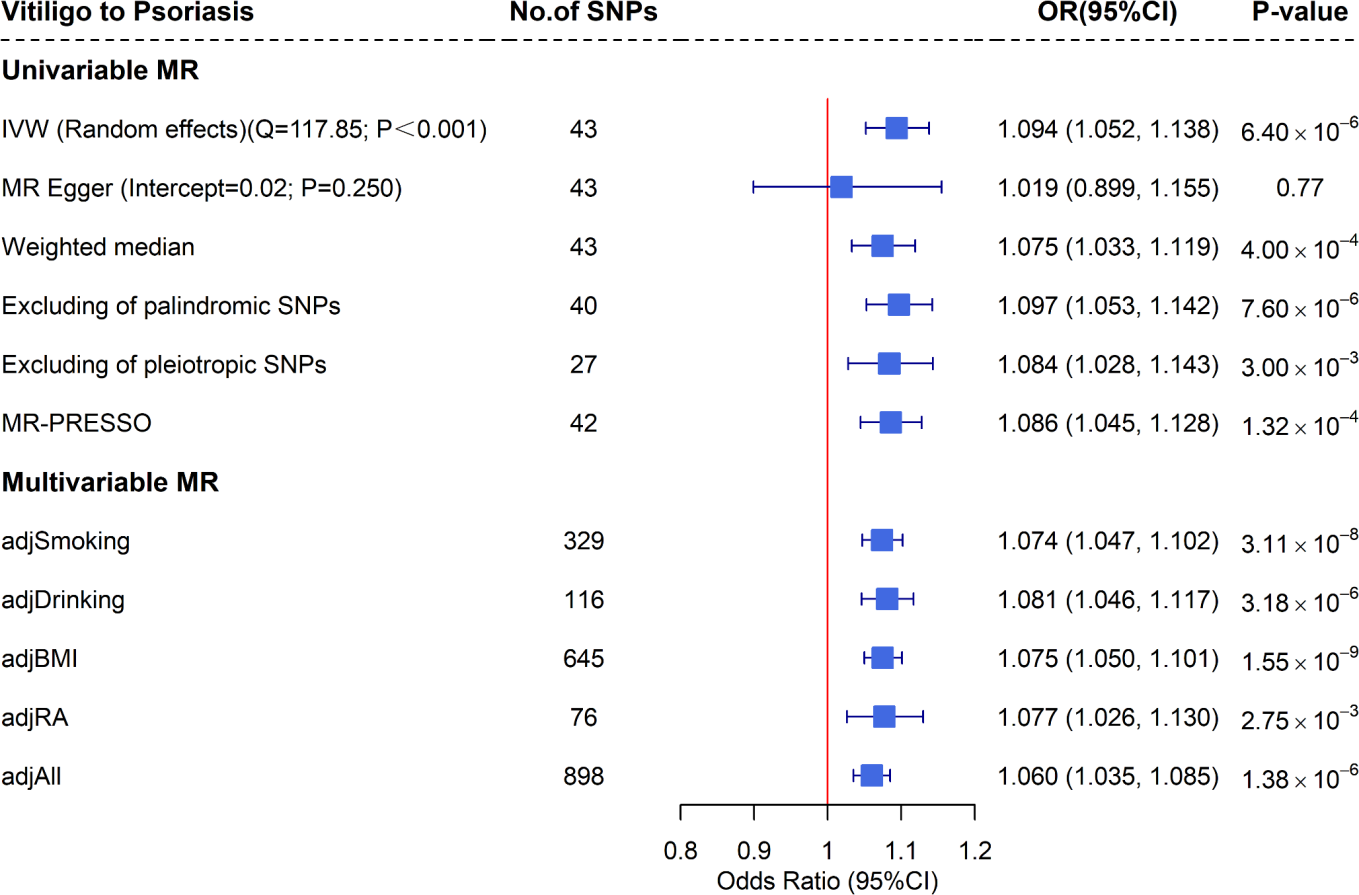
Estimates of the causal effect of vitiligo on psoriasis using univariable MR and multivariable MR. Abbreviations: SNP = Single-nucleotide polymorphism, OR = Odd ratio, CI = Confidence interval, MR = Mendelian randomization, IVW = Inverse variance weighted, MR-PRESSO = Mendelian Randomization Pleiotropy Residual Sum and Outlier, BMI = Body mass index, RA = Rheumatoid arthritis

The causal estimates of vitiligo on psoriasis are shown in Fig. 2. Since Cochran’s Q test (*Q* = 117.85, *P* < 0.001) suggested significant heterogeneity among the genetic IVs, the random effects IVW method was employed for the primary analysis. The results of the random-effects IVW method indicate a significant association between genetic predisposition to vitiligo and an elevated risk of psoriasis (*OR* = 1.094, 95% *CI*: 1.052, 1.138). Furthermore, the estimates were directionally consistent with IVW using MR-Egger regression (*OR* = 1.019, 95% *CI*: 0.899, 1.155), as well as with the weighted median method (*OR* = 1.075, 95% *CI*: 1.033, 1.119). No evidence of horizontal pleiotropy was detected (*P*_MR−Egger intercept_ = 0.250). Sensitivity analyses excluding pleiotropic SNPs (*OR* = 1.084, 95% *CI*: 1.028, 1.143), excluding palindromic SNPs (*OR* = 1.097, 95% *CI*: 1.053, 1.142), and applying the MR-PRESSO approach (*OR* = 1.086, 95% *CI*: 1.045, 1.128) yielded comparable results. In addition, the leave-one-out analysis indicated that the observed causal association was not driven by any single SNP, demonstrating the robustness of the results (Supplementary Fig. 1).

Further multivariate MR results showed that when adjusting for smoking (*OR* = 1.074, 95% *CI*: 1.047, 1.102), drinking (*OR* = 1.081, 95% *CI*: 1.046, 1.117), BMI (*OR* = 1.075, 95% *CI*: 1.050, 1.101), and RA (*OR* = 1.077, 95% *CI*: 1.026, 1.130) separately and for all confounders (*OR* = 1.060, 95% *CI*:1.035, 1.085) simultaneously, the observed causal associations remained statistically significant despite being somewhat attenuated, suggesting that vitiligo may have an independent causal role in psoriasis risk.

### The causal effect of psoriasis on vitiligo

**Fig. 3:**
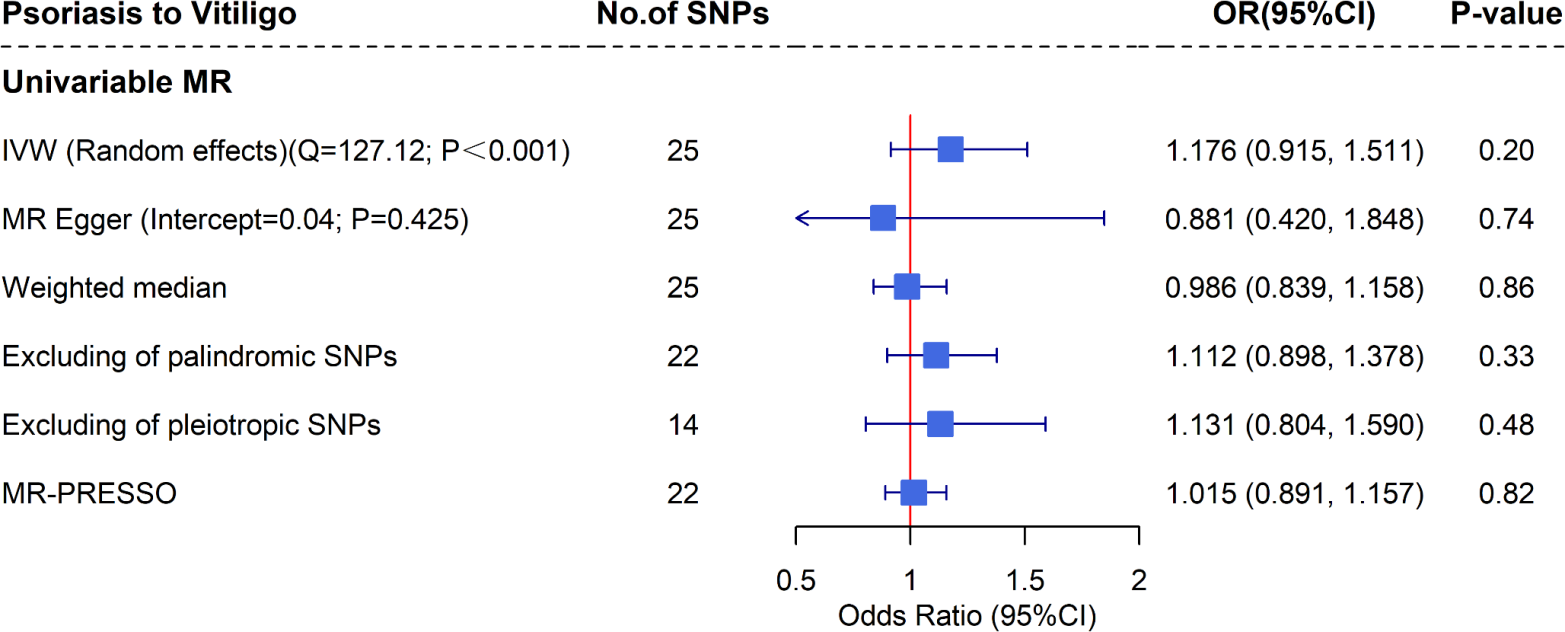
Estimates of the causal effect of psoriasis on vitiligo using univariable MR. Abbreviations: SNP = Single-nucleotide polymorphism, OR = Odd ratio, CI = Confidence interval, MR = Mendelian randomization, IVW = Inverse variance weighted, MR-PRESSO = Mendelian Randomization Pleiotropy Residual Sum and Outlier, BMI = Body mass index

The causal estimates of psoriasis on vitiligo are shown in Fig. 3. Using the random-effects IVW approach (*Q* = 127.12, *P* < 0.001), genetic liability to psoriasis was not associated with the risk of vitiligo (*OR* = 1.176, 95% *CI*: 0.915, 1.511), which remained consistent in MR-Egger regression (*OR* = 0.881, 95% *CI*: 0.420, 1.848) and weighted median approach (*OR* = 0.986, 95% *CI*: 0.839, 1.158). No evidence of horizontal pleiotropy was detected (*P*_MR−Egger intercept_ = 0.425). Sensitivity analyses, which excluded pleiotropic SNPs (*OR* = 1.131, 95% *CI*: 0.804, 1.590), palindromic SNPs (*OR* = 1.112, 95% *CI*: 0.898, 1.378), and utilized MR-PRESSO (*OR* = 1.015, 95% *CI*: 0.891, 1.157), as well as the leave-one-out analysis (Supplementary Fig. 2), all reported null effects.

## Discussion

By utilizing large-scale GWASs summary statistics, we performed a bidirectional two-sample MR study to evaluate the bidirectional relationship between vitiligo and psoriasis. We observed a significant and independent causal effect of genetic liability to vitiligo on psoriasis. However, there was insufficient evidence to support a causal effect of genetic liability to psoriasis on vitiligo.

We found a causal relationship between vitiligo and increased risk of psoriasis, which is supported by evidence from several previous observational studies [7, 11, 12]. For instance, a case-control study involving 74,415 participants found that those with vitiligo had a higher risk of developing psoriasis compared to participants without vitiligo [7]. Furthermore, another retrospective cohort that included 123,326 participants also found that vitiligo patients would have a 1.7-fold increased risk of secondary psoriasis [12]. However, it should be noted that a recent MR-based study reported a non-significant causal association between vitiligo and the risk of developing psoriasis [16], which is inconsistent with our results. One possible explanation for this difference in results is that the present study utilized GWAS summary data for psoriasis containing a larger sample size, obtaining more available SNPs (17 vs 43) [16]. This improves the statistical efficacy of causal inference, which in turn helps to identify potential causal relationships in the direction of vitiligo and psoriasis risk. In addition, compared with previous studies, the present study further revealed an independent causal effect of vitiligo on the risk of developing psoriasis after adjusting for potential confounders using multivariate MR, which extends previous studies.

The exact mechanisms underlying the association between vitiligo and the risk of psoriasis remain unknown. However, several potential mechanistic pathways have been previously proposed that may help explain the identified association. First, vitiligo and psoriasis share a common Th-1/Th-17 immune response pathway [35]. Previous studies have found that increases the main pro-inflammatory cytokines produced by Th-1 cells (e.g., interferon-gamma, tumor necrosis factor-α) and cytokines secreted by Th-17 cells (e.g., IL-17, IL-23) are observed in both diseases [35]. As these immune pathways are activated when vitiligo occurs, the persisted inflammatory environment can further induce psoriasis-associated immune responses, contributing to the development of psoriasis. Second, vitiligo and psoriasis share some common susceptibility genes (e.g., IFIH1, BTNL2) [36] and genetic loci (e.g., HLA-C/HLA-B rs9468925) [37]. When individuals with these susceptibility genes or genetic loci develop vitiligo, the combination of genes can lead to increased susceptibility to psoriasis. In addition, due to Koebner’s phenomenon, vitiligo leads to alterations in the skin’s barrier function and immune microenvironment following damage to the skin, such as leukoplakia, which may also induce psoriatic lesions [38]. Although these potential mechanisms explain to some extent the association of vitiligo with an increased risk of developing psoriasis, the specific mechanisms underlying the association remain unclear and require further study.

However, we had insufficient evidence to support a causal relationship between psoriasis and an increased risk of vitiligo, which is inconsistent with the results of some previous observational studies [11, 39]. The reasons for this inconsistency in results are unclear, but we speculated that it might be related to the following two reasons. On the one hand, positive associations found in previous observational studies may be due to unmeasured or unadjusted confounders. For instance, prior studies have shown that gut microbiota is associated with psoriasis and vitiligo [40]; however, conventional observational studies have not adjusted for this important factor, which may lead to spurious associations between both. On the other hand, the possibility of a causal relationship between psoriasis and an increased risk of vitiligo cannot be completely dismissed due to the limited statistical power of the MR analysis conducted in this study. Specifically, the 25 SNPs associated with psoriasis accounted for only 0.34% of the phenotypic variance in psoriasis. Moreover, the power evaluation revealed that the study had an 80% statistical power to detect a relative increase in vitiligo risk of more than 78% (i.e., *OR* = 1.78) (Supplementary Table 3). However, if the true causal effect of psoriasis on vitiligo is less than 78%, our study lacked the necessary statistical power to detect this potential causal relationship. Consequently, further investigations are required to validate the association between psoriasis and secondary vitiligo.

There are some limitations of this study that need to be noted. Firstly, despite the largely augmented set of IVs we used, these IVs only accounted for a modest portion of the phenotypic variance of each trait. Our study lacked adequate statistical power to detect a causal effect of psoriasis on vitiligo and future studies with larger sample sizes should be designed to replicate or dispute our findings. Secondly, the unavailability of individual data prevented us from conducting stratified analyses (e.g., age and sex) using the available summary statistics dataset. Finally, the GWAS summary data for vitiligo and psoriasis were obtained from populations of European ancestry, and generalization of these findings to other ethnic populations may be limited due to differences in the genetic structure (including gene frequencies, linkage disequilibrium patterns, and genetic heterogeneity) and the environments to which different ethnic populations are exposed (including diets, lifestyles, and environmental pollutants) [41]. Future research should examine the same topics in different racial or interracial populations to validate and expand the findings of current studies based on populations of European ancestry.

## Conclusion

In summary, based on evidence from two-sample MR studies, we identified a significant and independent causal role of vitiligo in the subsequent risk of psoriasis. However, whether psoriasis is causally associated with the risk of subsequent vitiligo is unknown, and further studies are needed to confirm it. Nonetheless, the results of this study emphasize that enhanced early screening for psoriasis among patients with vitiligo may improve the disease burden of psoriasis.

## Electronic supplementary material

Below is the link to the electronic supplementary material.

Supplementary Material 1

## Data Availability

All analyses were conducted using publicly available GWASs summary statistics. The GWAS for vitiligo is publicly available from https://ftp.ebi.ac.uk/pub/databases/gwas/summary_statistics/GCST004001-GCST005000/GCST004785/. The GWAS for psoriasis is publicly available from https://storage.googleapis.com/finngen-public-data-r9/summary_stats/finngen_R9_L12_PSORIASIS.gz.
