# Supplementary material for: Exploring the causal relationship between vitiligo and psoriasis: a bidirectional Mendelian randomization analysis
Source: Arch Dermatol Res. 2025 Mar 29;317(1):648. doi: 10.1007/s00403-025-04102-4 (PMC11954698; doi:10.1007/s00403-025-04102-4)
Supplement: Supplementary file 1 — Supplementary Material 1 [file 403_2025_4102_MOESM1_ESM.docx]

**Exploring the causal relationship between** **vitiligo and psoriasis: a bidirectional Mendelian randomization analysis**

**Zhengxing Xu, Chao Yang, Xuehui Gan, Peijing Yan, Changfeng Xiao, Yunli Ye ^*^, Xia Jiang ^*^**

*** Corresponding authors:**

**Yunli Ye**

School of Public Health, Southwest Medical University, No. 1 Section 1, Xianglin Road, Longmatan District, Luzhou, Sichuan, 646000, China.

Email: yunliye072@swmu.edu.cn

**Xia Jiang**

West China School of Public Health and West China Fourth Hospital, Sichuan University No.16, Section 3, South Renmin Road, Wuhou District, Chengdu 610041, China. Email: xiajiang@scu.edu.cn and [xia.jiang@ki.se](mailto:xia.jiang@ki.se).

**Supplementary Table 1.** Characteristics of 43 SNPs associated with vitiligo and their effect sizes with psoriasis.

**Supplementary Table 2.** Characteristics of 25 SNPs associated with psoriasis and their effect sizes with vitiligo.

**Supplementary Table 3.** Statistical power in bidirectional two-sample Mendelian randomization study of vitiligo and psoriasis.

**Supplementary Fig 1.** The leave-one-out analysis of the causal effect of vitiligo on psoriasis.

**Supplementary Fig 2.** The leave-one-out analysis of the causal effect of psoriasis on vitiligo.

**Supplementary Table 1.** Characteristics of 43 SNPs associated with vitiligo and their effect sizes with psoriasis.

| **SNPs** | **Chr** | **Position** | **EAF** | **A1** | **A2** | **Exposure (Vitiligo)** | | | | | **Outcome (Psoriasis)** | | |
| --- | --- | --- | --- | --- | --- | --- | --- | --- | --- | --- | --- | --- | --- |
|  |  |  |  |  |  | **Beta** | **SE** | **P** | **R^2^** | **F** | **Beta** | **SE** | **P** |
| rs10200159 | 2 | 55845109 | 0.06 | C | T | 0.41 | 0.05 | 3.73×10^-19^ | 3.00×10^-03^ | 133.19 | -0.01 | 0.04 | 8.21×10^-01^ |
| rs1031034 | 4 | 102223386 | 0.27 | A | C | -0.15 | 0.03 | 3.43×10^-08^ | 1.00×10^-03^ | 44.31 | 0.01 | 0.02 | 5.13×10^-01^ |
| rs1043101 | 11 | 35274829 | 0.41 | G | A | 0.21 | 0.02 | 5.26×10-^18^ | 3.00×10^-03^ | 133.19 | <0.01 | 0.02 | 9.10×10^-01^ |
| rs10774624 | 12 | 111833788 | 0.49 | A | G | -0.24 | 0.02 | 6.22×10^-23^ | 4.00×10^-03^ | 177.77 | -0.06 | 0.02 | 3.73×10^-05^ |
| rs10986311 | 9 | 127071493 | 0.37 | C | T | 0.14 | 0.02 | 1.01×10^-08^ | 1.00×10^-03^ | 44.31 | 0.05 | 0.02 | 8.06×10^-04^ |
| rs11021232 | 11 | 95320808 | 0.19 | C | T | 0.29 | 0.03 | 2.10×10^-23^ | 5.00×10^-03^ | 222.44 | -0.03 | 0.02 | 2.01×10^-01^ |
| rs11079035 | 17 | 40289012 | 0.17 | A | G | 0.19 | 0.03 | 6.77×10^-10^ | 1.00×10^-03^ | 44.31 | 0.03 | 0.02 | 7.62×10^-02^ |
| rs1126809 | 11 | 89017961 | 0.26 | A | G | -0.40 | 0.03 | 1.16×10^-43^ | 1.20×10^-02^ | 537.63 | 0.01 | 0.02 | 7.67×10^-01^ |
| rs117744081 | 7 | 29132279 | 0.03 | G | A | 0.61 | 0.06 | 8.72×10^-26^ | 4.00×10^-03^ | 177.77  . | 0.18 | 0.04 | 2.76×10^-06^ |
| rs12203592 | 6 | 396321 | 0.16 | T | C | -0.24 | 0.04 | 2.95×10^-10^ | 1.00×10^-03^ | 44.31 | -0.01 | 0.04 | 8.14×10^-01^ |
| rs12421615 | 11 | 64021605 | 0.35 | A | G | -0.14 | 0.03 | 4.81×10^-08^ | 1.00×10^-03^ | 44.31 | -0.06 | 0.02 | 2.75×10^-04^ |
| rs12482904 | 21 | 43851828 | 0.23 | A | T | 0.30 | 0.03 | 5.84×10^-29^ | 1.00×10^-02^ | 447.12 | -0.01 | 0.02 | 5.88×10^-01^ |
| rs12771452 | 10 | 115488331 | 0.26 | A | G | -0.20 | 0.03 | 4.43×10^-12^ | 2.00×10^-03^ | 88.71 | -0.03 | 0.02 | 7.39×10^-02^ |
| rs13076312 | 3 | 188089254 | 0.46 | T | C | 0.28 | 0.02 | 1.61×10^-30^ | 9.00×10^-03^ | 402.00 | 0.03 | 0.01 | 2.73×10^-02^ |
| rs1635168 | 15 | 28535266 | 0.07 | A | C | 0.31 | 0.04 | 8.78×10^-14^ | 3.00×10^-03^ | 133.19 | -0.01 | 0.07 | 9.24×10^-01^ |
| rs16843742 | 1 | 198672299 | 0.22 | C | T | -0.19 | 0.03 | 1.02×10^-09^ | 2.00×10^-03^ | 88.71 | -0.05 | 0.02 | 4.96×10^-03^ |
| rs2017445 | 12 | 56407072 | 0.34 | A | G | 0.29 | 0.02 | 6.62×10^-31^ | 5.00×10^-03^ | 222.44 | 0.05 | 0.02 | 2.74×10^-03^ |
| rs2111485 | 2 | 163110536 | 0.39 | A | G | -0.26 | 0.03 | 6.40×10^-25^ | 8.00×10^-03^ | 356.98 | -0.07 | 0.02 | 1.86×10^-05^ |
| rs2247314 | 6 | 167370230 | 0.33 | C | T | -0.24 | 0.03 | 1.72×10^-18^ | 3.00×10^-03^ | 133.19 | -0.02 | 0.02 | 1.40×10^-01^ |
| rs229527 | 22 | 37581485 | 0.43 | A | C | 0.28 | 0.02 | 1.14×10^-30^ | 6.00×10^-03^ | 267.19 | <0.01 | 0.02 | 8.25×10^-01^ |
| rs2304206 | 19 | 50168871 | 0.25 | A | G | -0.17 | 0.03 | 2.36×10^-09^ | 2.00×10^-03^ | 88.71 | -0.02 | 0.02 | 3.18×10^-01^ |
| rs231725 | 2 | 204740675 | 0.34 | A | G | 0.17 | 0.03 | 1.49×10^-10^ | 2.00×10^-03^ | 88.71 | -0.02 | 0.01 | 1.60×10^-01^ |
| rs2476601 | 1 | 114377568 | 0.10 | A | G | 0.32 | 0.04 | 1.21×10^-18^ | 3.00×10^-03^ | 133.19 | 0.03 | 0.02 | 1.81×10^-01^ |
| rs2687812 | 8 | 133931055 | 0.48 | A | T | 0.17 | 0.02 | 2.19×10^-13^ | 7.00×10^-03^ | 312.04 | 0.01 | 0.02 | 5.47×10^-01^ |
| rs301807 | 1 | 8484823 | 0.45 | A | G | 0.19 | 0.02 | 4.14×10^-15^ | 3.00×10^-03^ | 133.19 | 0.03 | 0.02 | 6.92×10^-02^ |
| rs34346645 | 3 | 71557945 | 0.41 | A | C | -0.22 | 0.03 | 7.99×10^-19^ | 4.00×10^-03^ | 177.77 | -0.03 | 0.02 | 8.44×10^-02^ |
| rs35161626 | 3 | 23512312 | 0.46 | I | D | -0.14 | 0.03 | 3.13×10^-08^ | 1.00×10^-03^ | 44.31 | -0.06 | 0.02 | 1.44×10^-04^ |
| rs35860234 | 13 | 43070206 | 0.27 | G | T | 0.16 | 0.03 | 4.76×10^-09^ | 1.00×10^-03^ | 44.31 | 0.03 | 0.02 | 4.31×10^-02^ |
| rs41342147 | 2 | 242407588 | 0.12 | A | G | -0.22 | 0.04 | 3.70×10^-09^ | 3.00×10^-03^ | 133.19 | 0.06 | 0.03 | 5.06×10^-02^ |
| rs4268748 | 16 | 90026512 | 0.27 | C | T | -0.34 | 0.03 | 2.88×10^-33^ | 1.30×10^-02^ | 583.02 | -0.01 | 0.02 | 4.40×10^-01^ |
| rs4308124 | 2 | 112010486 | 0.41 | C | T | 0.14 | 0.02 | 3.96×10^-09^ | 2.00×10^-03^ | 88.71 | -0.02 | 0.02 | 2.41×10^-01^ |
| rs4807000 | 19 | 4831878 | 0.40 | A | G | 0.19 | 0.02 | 1.94×10^-14^ | 2.00×10^-03^ | 88.71 | 0.01 | 0.02 | 5.14×10^-01^ |
| rs6012953 | 20 | 49123043 | 0.48 | G | A | 0.14 | 0.02 | 9.47×10^-09^ | 2.00×10^-03^ | 88.71 | 0.03 | 0.02 | 9.38×10^-02^ |
| rs6059655 | 20 | 32665748 | 0.07 | A | G | -0.49 | 0.05 | 1.04×10^-19^ | 4.00×10^-03^ | 177.77 | -0.01 | 0.05 | 7.91×10^-01^ |
| rs6583331 | 3 | 196347253 | 0.45 | A | T | -0.14 | 0.02 | 2.53×10^-08^ | 2.00×10^-03^ | 88.71 | -0.04 | 0.01 | 5.02×10^-03^ |
| rs706779 | 10 | 6098824 | 0.46 | C | T | -0.26 | 0.02 | 7.20×10^-27^ | 1.20×10^-02^ | 537.63 | -0.03 | 0.02 | 2.72×10^-02^ |
| rs71508903 | 10 | 63779871 | 0.20 | T | C | 0.17 | 0.03 | 6.93×10^-09^ | 1.00×10^-03^ | 44.31 | -0.01 | 0.02 | 6.31×10^-01^ |
| rs72928038 | 6 | 90976768 | 0.17 | A | G | 0.24 | 0.03 | 1.00×10^-14^ | 3.00×10^-03^ | 133.19 | -0.01 | 0.02 | 6.43×10^-01^ |
| rs78037977 | 1 | 172715702 | 0.13 | G | A | 0.28 | 0.03 | 6.74×10^-17^ | 3.00×10^-03^ | 133.19 | 0.06 | 0.02 | 1.43×10^-02^ |
| rs78521699 | 6 | 2908591 | 0.11 | G | A | -0.24 | 0.04 | 2.54×10^-08^ | 1.00×10^-03^ | 44.31 | -0.01 | 0.02 | 6.32×10^-01^ |
| rs8083511 | 18 | 60028655 | 0.19 | C | A | 0.19 | 0.03 | 2.81×10^-10^ | 2.00×10^-03^ | 88.71 | 0.02 | 0.02 | 2.52×10^-01^ |
| rs8192917 | 14 | 25102160 | 0.24 | C | T | 0.22 | 0.03 | 8.91×10^-16^ | 2.00×10^-03^ | 88.71 | -0.01 | 0.02 | 5.92×10^-01^ |
| rs9611565 | 22 | 41767486 | 0.23 | C | T | -0.24 | 0.03 | 3.13×10^-15^ | 3.00×10^-03^ | 133.19 | 0.01 | 0.02 | 5.98×10^-01^ |

Abbreviations: SNP = Single^-nu^cleotide polymorphism, Chr = Chromosome, EAF = Effect allele frequency, A1 = Effect allele, A2 = Reference allele, SE = Standard error, R^2^ = Proportion of variance in the phenotype explained by genetic variants.

**Supplementary Table 2.** Characteristics of 25 SNPs associated with psoriasis and their effect sizes with vitiligo.

| **SNPs** | **Chr** | **Position** | **EAF** | **A1** | **A2** | **Exposure (Psoriasis)** | | | | | **Outcome (Vitiligo)** | | |
| --- | --- | --- | --- | --- | --- | --- | --- | --- | --- | --- | --- | --- | --- |
|  |  |  |  |  |  | **Beta** | **SE** | ***P*** | **R^2^** | **F** | **Beta** | **SE** | ***P*** |
| rs10193310 | 2 | 60911776 | 0.25 | A | G | 0.13 | 0.02 | 9.34×10^-14^ | 1.52×10^-04^ | 55.51 | -0.04 | 0.03 | 2.85×10^-01^ |
| rs1250566 | 10 | 79286696 | 0.41 | A | G | -0.09 | 0.02 | 6.64×10^-09^ | 9.24×10^-05^ | 33.64 | 0.02 | 0.03 | 6.07×10^-01^ |
| rs13153019 | 5 | 177355217 | 0.27 | C | T | 0.10 | 0.02 | 6.67×10^-10^ | 1.05×10^-04^ | 38.12 | 0.00 | 0.03 | 9.18×10^-01^ |
| rs139298380 | 5 | 159291452 | 0.03 | A | G | 0.31 | 0.04 | 4.59×10^-14^ | 1.56×10^-04^ | 56.91 | -0.13 | 0.08 | 1.09×10^-01^ |
| rs16903065 | 8 | 128528218 | 0.12 | A | C | -0.14 | 0.02 | 5.01×10^-09^ | 9.39×10^-05^ | 34.19 | -0.15 | 0.04 | 6.45×10^-04^ |
| rs2021511 | 16 | 11251046 | 0.27 | T | C | -0.11 | 0.02 | 2.51×10^-10^ | 1.10×10^-04^ | 40.03 | -0.03 | 0.03 | 3.28×10^-01^ |
| rs2664280 | 10 | 73857586 | 0.45 | T | A | 0.09 | 0.01 | 6.37×10^-09^ | 9.26×10^-05^ | 33.72 | -0.05 | 0.03 | 7.56×10^-02^ |
| rs2769979 | 20 | 49909274 | 0.60 | C | T | -0.10 | 0.02 | 2.64×10^-10^ | 1.10×10^-04^ | 39.93 | -0.01 | 0.03 | 7.03×10^-01^ |
| rs28998802 | 17 | 27797882 | 0.18 | A | G | 0.16 | 0.02 | 7.83×10^-18^ | 2.03×10^-04^ | 74.01 | -0.01 | 0.04 | 7.47×10^-01^ |
| rs33980500 | 6 | 111592059 | 0.07 | T | C | 0.24 | 0.03 | 8.26×10^-19^ | 2.15×10^-04^ | 78.45 | 0.03 | 0.05 | 6.07×10^-01^ |
| rs34536443 | 19 | 10352442 | 0.03 | C | G | -0.28 | 0.05 | 2.73×10^-09^ | 9.72×10^-05^ | 35.37 | -0.08 | 0.07 | 2.74×10^-01^ |
| rs60600003 | 7 | 37342861 | 0.10 | G | T | 0.15 | 0.02 | 2.62×10^-10^ | 1.10×10^-04^ | 39.94 | -0.03 | 0.05 | 4.94×10^-01^ |
| rs653169 | 11 | 110130255 | 0.57 | G | A | -0.09 | 0.02 | 1.14×10^-08^ | 8.95×10^-05^ | 32.58 | -0.08 | 0.03 | 1.26×10^-02^ |
| rs6556423 | 5 | 159430181 | 0.64 | T | C | -0.18 | 0.02 | 3.05×10^-32^ | 3.84×10^-04^ | 139.75 | 0.01 | 0.03 | 7.63×10^-01^ |
| rs674451 | 6 | 137895651 | 0.34 | C | T | 0.13 | 0.02 | 1.29×10^-16^ | 1.88×10^-04^ | 68.48 | -0.01 | 0.03 | 7.83×10^-01^ |
| rs6916186 | 6 | 24708295 | 0.91 | G | A | -0.15 | 0.03 | 2.20×10^-08^ | 8.60×10^-05^ | 31.31 | 0.01 | 0.05 | 8.66×10^-01^ |
| rs7310615 | 12 | 111427245 | 0.59 | G | C | -0.09 | 0.02 | 9.58×10^-09^ | 9.04×10^-05^ | 32.93 | -0.22 | 0.03 | 2.90×10^-14^ |
| rs74817271 | 5 | 151090412 | 0.07 | A | G | 0.22 | 0.03 | 4.12×10^-17^ | 1.94×10^-04^ | 70.73 | -0.06 | 0.06 | 3.10×10^-01^ |
| rs7542079 | 1 | 24968116 | 0.56 | C | T | 0.10 | 0.02 | 2.87×10^-10^ | 1.09×10^-04^ | 39.77 | 0.04 | 0.03 | 1.35×10^-01^ |
| rs76741620 | 11 | 36399020 | 0.06 | G | A | 0.17 | 0.03 | 1.06×10^-08^ | 8.99×10^-05^ | 32.74 | -0.13 | 0.09 | 1.57×10^-01^ |
| rs78456138 | 2 | 162275836 | 0.02 | T | C | -0.36 | 0.06 | 6.61×10^-10^ | 1.05×10^-04^ | 38.14 | -1.05 | 0.17 | 5.97×10^-10^ |
| rs80174646 | 1 | 67242472 | 0.05 | T | G | -0.23 | 0.04 | 2.81×10^-09^ | 9.70×10^-05^ | 35.31 | -0.04 | 0.06 | 4.77×10^-01^ |
| rs847 | 5 | 132660977 | 0.63 | C | T | 0.13 | 0.02 | 8.63×10^-16^ | 1.78×10^-04^ | 64.73 | 0.08 | 0.04 | 3.01×10^-02^ |
| rs8904 | 14 | 35402011 | 0.39 | A | G | -0.13 | 0.02 | 2.83×10^-16^ | 1.84×10^-04^ | 66.93 | -0.01 | 0.03 | 6.53×10^-01^ |
| rs9346778 | 6 | 159103761 | 0.16 | T | C | -0.12 | 0.02 | 1.55×10^-08^ | 8.79×10^-05^ | 31.99 | -0.03 | 0.04 | 3.43×10^-01^ |

Abbreviations: SNP = Single-nucleotide polymorphism, Chr = Chromosome, EAF = Effect allele frequency, A1 = Effect allele, A2 = Reference allele, SE = Standard error, R^2^ = Proportion of variance in the phenotype explained by genetic variant.

**Supplementary Table 3.** Statistical power in bidirectional two-sample Mendelian randomization study of vitiligo and psoriasis.

| Exposure | Outcome | No. of SNPs | R^2^ (%) | Proportion of cases (%) | Minimum detectable OR **^a^** |
| --- | --- | --- | --- | --- | --- |
| Vitiligo | Psoriasis | 43 | 16.20 | 2.48 | 0.93/1.07 |
| Psoriasis | Vitiligo | 25 | 0.34 | 10.57 | 0.33/1.78 |

^a^ Minimum detectable OR was calculated based on 80% power.

Abbreviations: SNP = Single-nucleotide polymorphism, R^2^ = Proportion of variance in the phenotype explained by genetic variants, OR = Odd ration.


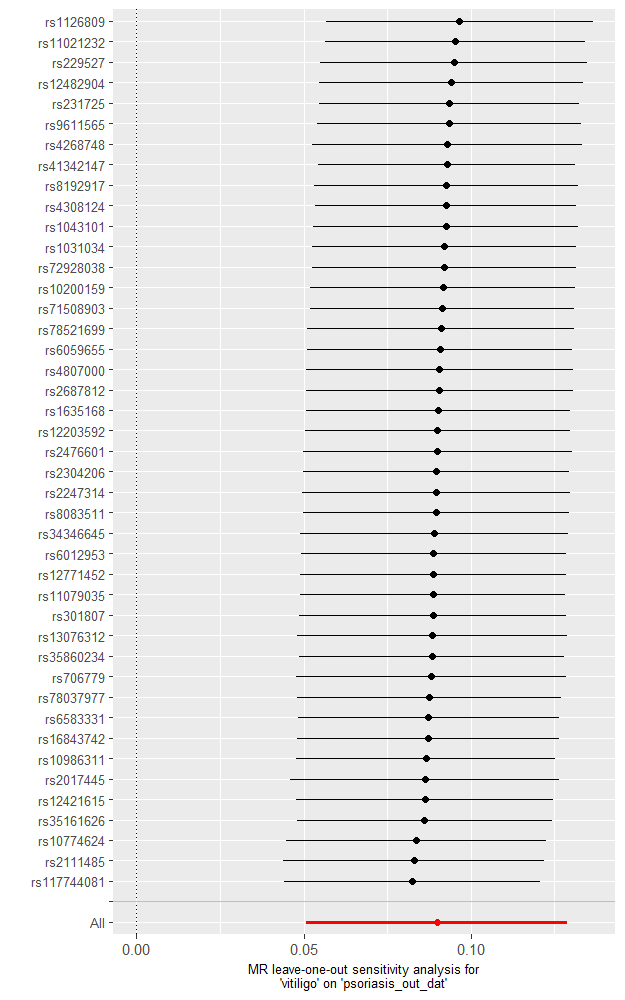


**Supplementary Fig 1.** The leave-one-out analysis of the causal effect of vitiligo on psoriasis.

**
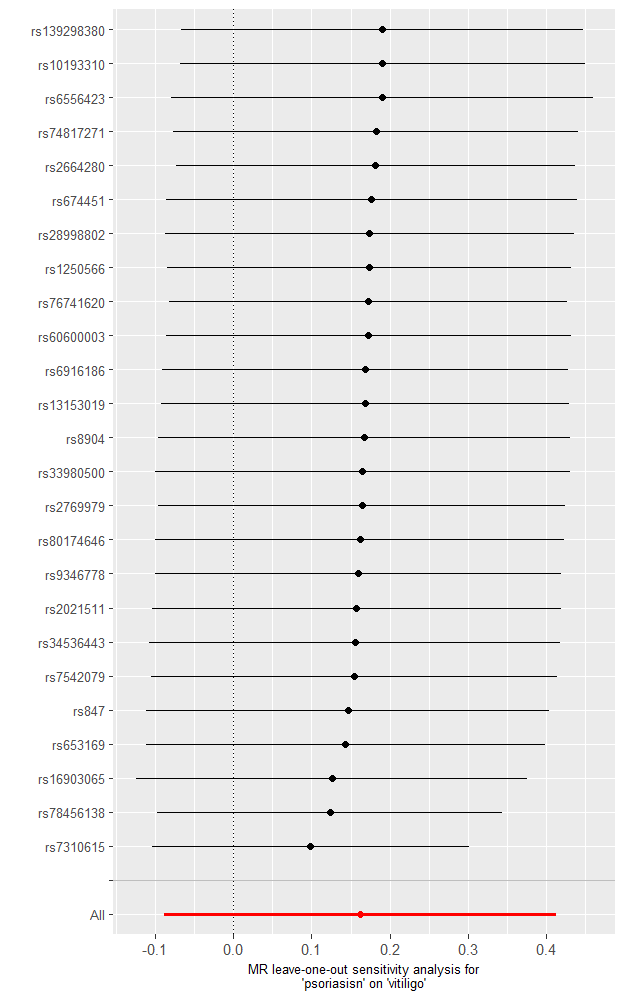
**

**Supplementary Fig 2.** The leave-one-out analysis of the causal effect of psoriasis on vitiligo.
